# A Mobile Health App Informed by the Multi-Process Action Control Framework to Promote Physical Activity Among Inactive Adults: Iterative Usability Study

**DOI:** 10.2196/59477

**Published:** 2025-04-23

**Authors:** Heather Hollman, Wuyou Sui, Haowei Zhang, Ryan E Rhodes

**Affiliations:** 1 Behavioural Medicine Lab School of Exercise Science, Physical and Health Education University of Victoria Victoria, BC Canada; 2 School of Kinesiology Western University London, ON Canada; 3 Behavioural Medicine Lab School of Exercise Science, Physical & Health Education University of Victoria Victoria, BC Canada

**Keywords:** physical activity, mobile apps, mobile health, mHealth, usability study, inactive adults, smartphone

## Abstract

**Background:**

Mobile health apps have high potential to address the widespread deficit in physical activity (PA); however, they have demonstrated greater impact on short-term PA compared to long-term PA. The multi-process action control (M-PAC) framework promotes sustained PA behavior by combining reflective (eg, attitudes) and regulatory (eg, planning and emotion regulation) constructs with reflexive (eg, habits and identity) constructs. Usability testing is important to determine the integrity of a mobile health app’s intrinsic properties and suggestions for improvement before feasibility and efficacy testing.

**Objective:**

This study aimed to gather usability feedback from end users on a first and a second version of an M-PAC app prototype.

**Methods:**

First, 3 workshops and focus groups, with 5 adult participants per group, were conducted to obtain first impressions of the M-PAC app interface and the first 3 lessons. The findings informed several modifications to the app program (eg, added cards with reduced content) and its interface (eg, created a link placeholder image and added a forgot password feature). Subsequently, a single-group pilot usability study was conducted with 14 adults who were not meeting 150 minutes per week of moderate-to-vigorous PA. They used the updated M-PAC app for 2 weeks, participated in semistructured interviews, and completed the Mobile App Usability Questionnaire (MAUQ) to provide usability and acceptability feedback. The focus groups and interviews were recorded, transcribed, and analyzed with content analysis informed by usability heuristics. The MAUQ scores were analyzed descriptively.

**Results:**

Participants from the workshops and focus groups (mean age 30.40, SD9.49 years) expressed overall satisfaction with the app layout and content. The language was deemed appropriate; however, some terms (eg, self-efficacy) and acronyms (eg, frequency, intensity, time, and type) needed definitions. Participants provided several recommendations for the visual design (eg, more cards with less text). They experienced challenges in accessing and using the help module and viewing some images, and were unsure how to create or reset the password. Findings from the usability pilot study (mean age 41.38, SD12.92 years; mean moderate-to-vigorous PA 66.07, SD57.92 min/week) revealed overall satisfaction with the app layout (13/13, 100%), content (10/13, 77%), and language (7/11, 64%). Suggestions included more enticing titles and additional and variable forms of content (eg, visual aids and videos). The app was easy to navigate (9/13, 69%); however, some errors were identified, such as PA monitoring connection problems, broken links, and difficulties entering and modifying data. The mean MAUQ total and subscale scores were as follows: total=5.06 (SD1.20), usefulness=4.17 (SD1.31), ease of use=5.36 (SD1.27), and interface and satisfaction=5.52 (SD1.42).

**Conclusions:**

Overall, the M-PAC app was deemed usable and acceptable. The findings will inform the development of the minimum viable product, which will undergo subsequent feasibility testing.

## Introduction

### Background

Regular physical activity (PA) is associated with reduced risk of cardiovascular disease, hypertension, diabetes, breast and colon cancer, and mortality [1]. It also has positive effects on cognition and mental health [2,3]. Unfortunately, PA rates worldwide are insufficient to reap these mental and physical health benefits [4]. Given the widespread burden of global inactivity, scalable PA interventions are needed. Mobile health (mHealth) apps have great potential for scalability and to meet harder-to-reach populations, such as rural residents, who are also at increased risk of inactivity [5]. As of 2024, >60% of the global population owns a smartphone, with even greater rates in high-income nations such as the United Kingdom (82%), the United States (82%), and Canada (72%) [6]. People spend the majority (88%) of their mobile time using apps [7], and in 2019, the average individual used 9 mobile apps per day and 30 apps per month [8].

mHealth apps are an appealing medium for PA behavioral interventions because they can provide up-to-date educational material while concurrently enabling continuous PA tracking and feedback [9-11]. They can also facilitate connections with peers and health providers [9-11] and more easily implement personalization and associations (eg, reminders) compared to print or in-person interventions [12]. From a theoretical standpoint, they can deliver a higher amount of behavior change techniques (BCTs) compared to in-person interventions [12]. Using well-defined BCTs (eg, goal setting and self-monitoring) has been associated with increased PA intervention engagement, which is an essential metric to address given the high app attrition rate and the connection between engagement and resulting PA behavior [13-15].

In line with the additional BCTs and related features, mHealth apps have demonstrated promising potential to impact PA behavior. In a recent meta-analysis of PA mHealth apps or trackers with automated and continuous self-monitoring and feedback, Laranjo et al [16] reported a small-to-moderate effect on PA (standardized mean difference [SMD]=0.350; 95% CI 0.236-0.465; *I*^2^=69%; *T*^2^=0.051) with a mean follow-up of 13 weeks. These standardized effects are higher than meta-analyses of workplace PA interventions (SMD=0.21) [17], internet-delivered PA interventions (SMD=0.14) [18], and face-to-face PA interventions (SMD=0.29) [19] and are equivalent to the 85th percentile of benchmarked PA intervention effects [20]. PA mHealth app interventions have also demonstrated longer-term (>6 months) effects on PA; however, the effect sizes tend to decrease over time [21], similar to PA interventions in general [22]. Because the health benefits can only be accrued if PA behavior change is sustained, PA maintenance, in addition to engagement, should be a focus of future PA mHealth apps.

To determine intervention components needed for longer-term PA behavior, PA theorists have attempted to conceptualize the acts of PA behavior adoption and maintenance separately [23,24]. For example, although constructs of traditional PA theories in the social cognitive domain (eg, outcome expectations or action planning) seem to be moderate predictors of PA adoption, they may be less comprehensive for maintenance [24,25]. Social cognitive theories highlight the importance of forming PA intentions; however, these approaches sometimes fail to account for the intention-PA gap (ie, the proportion of individuals who do not follow through with regular PA behavior that aligns with their PA intentions) [26-28]. A recent meta-analysis showed that 48% of adult PA intenders fail to follow through with PA [29].

Several theoretical approaches have been developed to close the intention-behavior gap [27,30]. An example is the multi-process action control (M-PAC) framework, which combines reflective processes, such as those in traditional social cognitive approaches (eg, attitudes), with regulatory (eg, self-monitoring and emotion regulation) and reflexive (eg, habit and exercise identity) processes to facilitate PA behavior change [31]. Collectively, the M-PAC constructs have a causal structure that begins with intention formation, progresses to the adoption of action control, and ultimately leads to sustained action control. Each stage is interconnected, with processes that are mutually reinforcing and reciprocal [32]. Each construct independently provides meaningful and significant contributions to translating positive intentions to PA behavior, with the largest predictive effect sizes coming from the latter reflexive processes [31]. PA interventions (in-person and web-based) informed by the M-PAC framework have demonstrated promising results on PA behavior and PA maintenance, promoting constructs of habit, self-identity, and behavioral regulation in feasibility trials [33-36]. However, an mHealth app informed by the M-PAC framework is yet to be tested.

To advance the likelihood of a successful and scalable mHealth app, it is recommended to assess its intrinsic properties, including usability and feasibility, in the initial prototyping phases [37]. An initial focus on these intrinsic properties can ultimately lead to greater attribution of the extrinsic metrics and outcomes to the intervention. Usability is the assessment of the quality of the interaction between the user and the intervention, while feasibility is the assessment of the ability of the intervention to work as intended in a given context [37]. Usability testing can help to uncover the overall interest in and eagerness to use the mHealth app, potential impediments to its use, and suggestions for improvement or innovation [38]. This usability phase is usually iterative, whereby prototypes are refined and optimized through several stages based on user feedback. It is also essential to involve end users to ensure that the intervention is relevant and meets the specific needs and preferences of the target population [39,40]. Once this phase is complete, a minimum viable product (MVP) can be built and then pilot-tested to test for further usability and acceptability, followed by evaluation of its efficacy [38].

### Research Objective

Thus, the objective of this research was to gather user feedback on the first and second prototypes of the M-PAC app to inform the design and development of the MVP. We initially explored the first impressions of the M-PAC app prototype using focus group methodology to gather important preliminary feedback from a group with diverse views and perspectives [41]. Subsequently, we used a usability pilot study [42] to retrieve more in-depth feedback on the usability and acceptability of the M-PAC app from potential users—individuals who intend to exercise but are not currently meeting the recommended PA guidelines.

## Methods

### Overarching App Development Framework

The M-PAC app development and design were in accordance with the IDEAS (integrate, design, assess, and share) framework [38]. Specifically, this study described 2 iterative rounds of phases 5 (prototype potential products) and 6 (gather user feedback) [38]. As per the IDEAS framework phase 5, an initial prototype was assessed during a series of workshops and focus groups to gather first impressions and suggestions for improvement. Commensurate with phase 6 of the IDEAS framework, the suggestions from the workshops and focus groups were implemented into the M-PAC app, and subsequently, the updated second app prototype was assessed in a usability pilot study, in which a new group of participants had the opportunity to use the app as intended for a few weeks and then provide additional feedback in individual interviews.

Phase 1 (workshops and focus groups) and the qualitative interview component of phase 2 (usability pilot) were reported following the COREQ (Consolidated Criteria for Reporting Qualitative Research) criteria [43]. Phase 2 (usability pilot) was reported following a modified version of the CONSORT (Consolidated Standards of Reporting Trials) guidelines [44], as recommended by Lancaster and Thabane [45] for nonrandomized pilot studies.

### Ethical Considerations

These studies received ethics approval from the University of Victoria Human Research Ethics Board (21-0248; 21-0360). All participants provided informed consent before participating in the studies, and all study data are anonymous. Participants in phase 2 (usability pilot) received a CAD $15 (US $10.88) honorarium for participating in the study.

### Phase 1: Workshops and Focus Groups

#### Design

Online hands-on workshops followed by focus groups were conducted. The workshops were conducted between November 2021 and March 2022. Focus groups were designed to be run with 4 to 6 participants, with a total of 15 to 20 participants, based upon previous usability studies using apps and web-based delivery [46-49]. The workshops and focus groups were led by WS and supported by HH (who also took field notes). WS has a PhD in health psychology, was a postdoctoral fellow at the time of the study, and identifies as a man. HH has a master’s degree in rehabilitation science, was a PhD student at the time of the study, and identifies as a woman. WS had prior experience in conducting research focus groups and interviews and HH did not. WS and HH both have prior experience analyzing qualitative data. WS and HH introduced themselves to the participants as research trainees with a focus on PA behavior change.

#### Participants

Eligible participants were adults aged 19 to 64 years who owned a smartphone with either an iOS or Android operating system and were fluent in English. Participants were recruited via online advertisements on the research laboratory’s public X account (X Corp; @bmedlab); public Instagram account (Meta Platforms, Inc; @uvicbmed); public community Facebook (Meta Platforms, Inc) groups local to Victoria, British Columbia; and through snowball sampling. All communication between researchers and participants took place over email. Some participants were known to the researchers because they worked in the same university; however, none of the participants had provided feedback on the app before the study.

#### Procedures

Interested participants contacted the researchers via email and were sent an email with the letter of information and consent form. Upon providing consent, participants were asked to provide availability for a 60-minute workshop and focus group. Participants were informed that they would be given a pseudonym during the workshops and focus groups to protect their identity. Participants were later contacted to reconfirm their availability once 4 to 5 participants reported having a mutually available time and were sent a Zoom videoconferencing link upon confirmation (Zoom Video Communications). In addition, participants were asked to download the Pathverse app platform [50] (Pathverse, Inc), which gave them access to the M-PAC app on their smartphone, to be able to actively participate in the workshop portion of the session.

One day before the date of the workshop or focus group, participant emails were used to create profiles on the admin portal of Pathverse, so that participants would have access to the M-PAC app. On the arranged time of the workshop or focus group, participants joined into a Zoom call with 2 researchers. All participants were given pseudonyms and were unable to turn on video. The lead researcher shared their smartphone screen and led the workshop and focus group discussion, while the other researcher managed the video recording software and any questions within the Zoom chat. Upon entering the Zoom call, the lead researcher asked participants to open the Pathverse app and then instructed participants on how to create a profile on Pathverse using their email. Once all the participants had access to the app, the lead researcher began a walkthrough of the app.

Participants were first shown how to access the lessons, along with a brief overview of how to navigate through lesson cards, the different types of cards, and how to mark a lesson as complete, using “Lesson 0: Tutorial” and “Lesson 1: What is Physical Activity” as examples. Afterward, participants were directed to the tracking features on the app. Participants were shown how to sync their step count to the app as well as how to track and log PA, weight, and blood pressure. Participants were then shown the goal-setting feature of the app. The lead researcher created an example goal, demonstrating to participants how to name the goal, set priority and reminders on the goal, and how to mark goals as completed. Finally, participants were shown how to navigate to the home page of the app and to find the in-app navigation tutorial if they got stuck. Participants were then given 5 to 10 minutes to explore the app and its features independently. Overall, the workshop portion of the session took approximately 25 to 30 minutes.

Following the workshop, participants were informed that they would be starting the focus group portion of the session. The lead researcher read the introductory script and then prompted participants to ask any questions before beginning with the list of semistructured questions (Multimedia Appendix 1). The focus group portion of the session took approximately 25 to 30 minutes. The workshop and focus group guide was pilot-tested among the research team. Only the participants and researchers were present during the workshops and focus groups. There were no repeat workshops and focus groups.

#### Intervention: The M-PAC App

**Figure 1:**
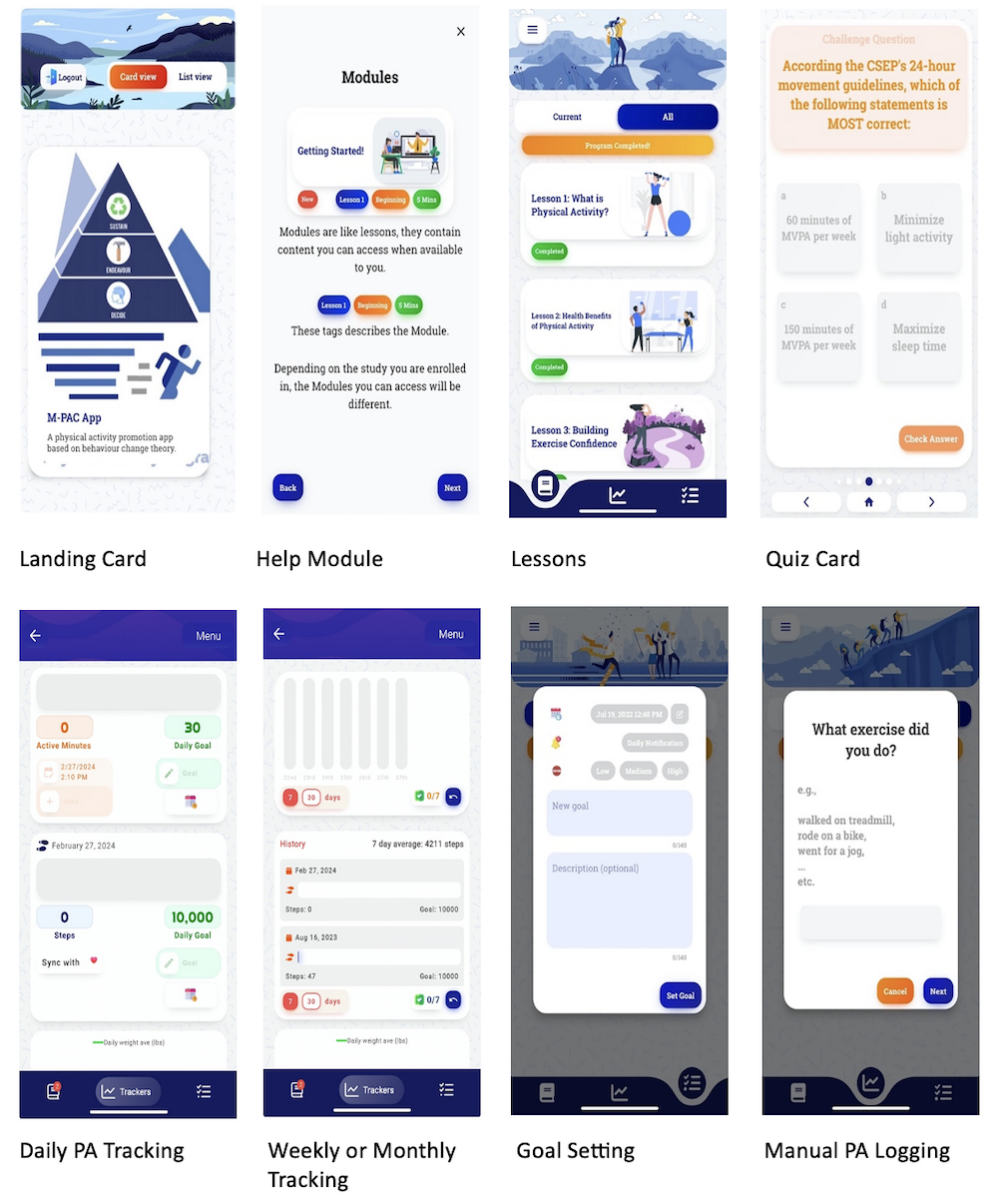
Screenshots of a physical activity mobile health app informed by the multi-process action control (M-PAC) framework (the M-PAC app). PA: physical activity.

The M-PAC app is an evidence-based mobile app designed to support users in changing their PA behavior, developed by researchers specializing in PA behavior. The app is built upon the Pathverse app platform [50]. Pathverse is an mHealth development platform designed for health and wellness professionals and researchers. This platform consists of an admin web-portal interface and a participant Pathverse app (available in both IOS and Android stores). The M-PAC app, specifically, includes educational content in the form of lessons; self-monitoring features, such as step tracking and PA logging; and a goal-setting feature, whereby users can set goals for themselves as well as review completed goals (Figure 1).

Lessons within the app are broken down into individual “cards,” which users navigate by swiping left or right, or via the arrow buttons at the bottom of the screen. Cards present either (1) a brief block of text, (2) a brief block of text with an image placed below or above, (3) a brief block of text with a link to an external web page, or (4) a challenge question. The challenge question card presents the user with a short question and 4 possible answers. Selecting any answer presents the user with a new slide indicating whether their answer was correct or incorrect, as well as a brief explanation for the correct answer.

Lessons within the app are scaffolded according to the different constructs within the M-PAC framework. Lessons 1 to 4 are designed to address reflective processes of the M-PAC framework: instrumental attitudes (“What are the health benefits of PA?”), perceived opportunity (“Do I have the resources to do PA?”), perceived capability (“Am I confident I can perform this PA?”), and affective judgments (“Do I like PA?”). Similarly, lessons 5 to 10 are aimed at addressing the regulatory (ie, skills important in facilitating continued PA behavior, such as goal setting and planning) and reflexive processes (eg, habit and exercise identity) of the M-PAC. In addition, lessons contain challenge cards that assess users’ understanding of the content within their current lesson. Refer to Multimedia Appendix 2 [51] for a full list of app lessons, mechanisms of action, BCTs, and behavior change ontologies applied [52,53]. Lessons must be completed in order (ie, lesson 0, lesson 1, lesson 2, etc) to unlock subsequent lessons, but completed lessons can be revisited once unlocked. Given the usability focus of this study, only lessons 1 to 3 (including a lesson 0: tutorial) were presented to participants.

#### Outcomes

The primary outcome of this phase was qualitative feedback on the usability of the M-PAC app drawn from the focus groups. Specifically, questions within the focus group were drawn from Nielsen's 10 usability heuristics for user interface design [54]. These heuristics were adapted for this work to guide the structured focus group questions, which addressed the following: layout and information, appropriateness of language, backing out or undoing an action, consistency in language and features, errors and mistakes, forgetting acronyms, efficiency of interface and navigation, visual design, ease in diagnosing and resolving errors, and help and documentation. Probing questions for each main question were also planned if group responses were vague or open ended. For example, the first main question was, “How did you find the layout and information of the app?” with probing questions of “If intuitive, what made the layout or information intuitive?” and “If unintuitive, what made the layout or information unintuitive? What would be more helpful?” The specific questions related to these heuristics can be found in Multimedia Appendix 3. Participant responses were recorded using the video recording software Open Broadcast Software (OBS) Studio [55] (OBS Studio Contributors).

Participant demographics of age, gender, and highest attained education were assessed using single-item questions. Furthermore, the brand of smartphone owned, ability to use the features of a smartphone, ability to engage with other technologies, and whether a PA app has been downloaded or used in the past were also assessed using single-item measures.

#### Analysis

Focus groups were recorded using OBS Studio and transcribed verbatim [55]. Transcripts were coded and analyzed thematically, according to the 10 usability heuristics [54] adapted for this study, using NVivo (QSR International) [56]. Participants were not asked to provide feedback on the transcripts or findings. Two coders independently coded the transcripts, with conflicts being resolved by a third coder.

### Phase 2: Usability Pilot

#### Design

A 2-week, single-group pilot study was conducted virtually between August 2022 and February 2023. The researchers were located in Victoria, British Columbia, Canada, and the participants were located anywhere in Canada.

#### Participants

Eligible participants were Canadian adults aged 19 to 64 years who owned a smartphone device with either an iOS or Android operating system, were fluent in English, and reported engaging in <150 minutes of moderate-to-vigorous PA (MVPA) per week. Individuals were excluded if they participated in the phase 1 workshops or focus groups or were involved in the design and development of the M-PAC app. Participants were recruited via online advertisements on the research laboratory’s public X account (@bmedlab), public Instagram account (@uvicbmed), and public community Facebook groups local to Victoria, British Columbia; with paid Facebook advertisements; and snowball sampling. All communication between researchers and participants took place over email. A total of 10 to 15 participants was deemed appropriate based on previous usability testing studies examining apps [57,58].

#### Procedures

Interested individuals contacted the research team via email and were sent an email with the letter of information and a consent form. Upon agreeing to participate in the study, participants were sent instructions on how to download the M-PAC app, as well as a demographics questionnaire. Participants also booked a 30-minute baseline meeting with a researcher to walkthrough the M-PAC app over Zoom. During the baseline meeting, participants were given a walkthrough of the M-PAC app, identical to the workshop from phase 1. Upon completing the walkthrough, participants were asked to schedule a 30-minute exit interview in 2 weeks and finish lessons 1 to 3 within those 2 weeks.

During the exit interview, the researcher followed the semistructured interview guide to assess participants’ experiences with the M-PAC app (Multimedia Appendix 3). The interview guide was pilot-tested among the research team. Interviews were conducted by WS over Zoom, audio recorded using OBS Studio, and lasted 20 to 30 minutes. Only the participants and researchers were present, and there were no repeat interviews. After completing the exit interview, participants were told that they would be sent the Mobile App Usability Questionnaire (MAUQ) to their email in 24 hours, and to complete the MAUQ within 3 days of receiving the questionnaire [59]. Upon completion of the MAUQ, participants were given a CAD $15 (US $11.88) honorarium. There were no changes made to the methods upon pilot study commencement.

#### Intervention

Following the completion of phase 1, several modifications were made to the M-PAC app. Changes to content included the following: (1) modifying the introductory card for the self-efficacy lesson from “Introduction to self-efficacy...” to “Ways to improve confidence (also known as self-efficacy)...”, (2) ensuring all acronyms (eg, MVPA; Canadian Society for Exercise Physiology; frequency, intensity, time, and type [FITT]; and Get Active Questionnaire) were clearly defined, (3) bolding some terms to highlight them (eg, FITT), (4) reducing the amount of text on cards and increasing the number of cards per lesson, and (5) adding a references module and providing links to references from each lesson. Changes to the Pathverse platform included the following: (1) optimizing image loading times, (2) creating an external link placeholder image that reads “Link,” and (3) adding a “Forgot your password?” feature. The updated version of the M-PAC app with the aforementioned modifications was used for the usability pilot study.

#### Outcomes

The primary outcome of this phase was qualitative feedback on the usability and satisfaction of the M-PAC app, gathered through the exit interview. Probing questions for each main question were also planned if participants’ responses were vague or open ended. For example, for the question, “Can you tell me what you liked best about the M-PAC app?”, probing questions of “Were there any features in particular?” and “What about the content and/or layout?” were planned. The specific questions within this interview guide can be found in Multimedia Appendix 3. Participant responses were recorded using the video recording software OBS Studio [55]. The a priori criteria for moving on to a randomized pilot trial was an overall consensus that the intervention was usable (MAUQ score≥5), and at least 60% (8/13) of participants were satisfied with the intervention, with minor to moderate suggestions for improvement.

Quantitative feedback on the usability of the M-PAC app was collected via the MAUQ [59], specifically the version for stand-alone mHealth apps. This version of the MAUQ is an 18-item self-report questionnaire that assesses an mHealth app’s ease of use, interface and satisfaction, and usefulness. The questionnaire demonstrated construct and criterion validity and reliability, with Cronbach α=0.91 for the total MAUQ, 0.71 for ease of use, 0.93 for interface and satisfaction, and 0.65 for usefulness internal consistency.

Participant demographics of age, gender, and highest attained education were assessed using single-item questions. Furthermore, the brand of smartphone owned, ability to use the features of a smartphone, ability to engage with other technologies, and whether a PA app has been downloaded or used in the past were also assessed using single-item measures. Participants’ average weekly PA was assessed using the modified Godin Leisure-Time PA questionnaire [60]. The estimated weekly MVPA was calculated by multiplying the frequency and duration of moderate- and vigorous-intensity PA minutes and adding them together. The Godin-Leisure-Time PA questionnaire has demonstrated acceptable validity and reliability in a variety of settings, populations, and countries [60,61]. There were no changes made to the assessments or measurements after the trial commenced.

#### Analyses

Exit interviews were recorded using OBS Studio and transcribed verbatim. Transcripts were coded and analyzed thematically, according to the 10 usability heuristics [54] adapted for this study, using NVivo [56]. Participants were not asked to provide feedback on the transcripts or findings. Two coders independently coded the transcripts, with conflicts being resolved by a third coder.

Descriptives (ie, mean and SD) for quantitative usability feedback from the MAUQ were calculated for total MAUQ scores, as well as for the ease of use, interface and satisfaction, and usefulness subscales [59].

## Results

### Phase 1: Workshops and Focus Groups

#### Participants

We conducted 3 focus groups of 5 participants each. Participants were primarily women (13/15, 87%) with a mean age of 30.4 (SD 9.49) years. Most had at least a college or university degree (14/15, 93%) and self-reported having above average abilities to use the functions of their smartphone (11/15, 73%), and just under half had previously downloaded a mobile app to help increase their PA (7/15, 47%; eg, Down Dog, Fitbit, Health, Nike Training Club app, Endomondo, and Sweat app). The most common brand of smartphone was Apple iPhone (9/15, 60%), followed by Samsung (4/15, 27%) and Google Pixel (2/15, 13%; Table 1). None of the participants refused to participate or dropped out of the study.

**Table 1:** Baseline characteristics of the workshops, focus groups, and pilot study participants.

			Workshops and focus groups (n=15)	Pilot (n=14)^a^
Age (y), mean (SD)			30.4 (9.49)	41.38 (12.92)
**Gender, n (%)**				
	Women		13 (87)	11 (85)
	Men		2 (13)	2 (15)
British Columbia			—^b^	Yes=8 (62) and no=5 (38)
Country (Canada)			15 (100)	13 (100)
**Highest education, n (%)**				
	High-school diploma		1 (7)	1 (8)
	Vocational school or some college		0 (0)	3 (23)
	College or university degree		6 (40)	5 (38)
	Professional or graduate degree		8 (53)	4 (31)
**Brand of smartphone, n (%)**				
	Google Pixel		2 (13)	1 (8)
	Samsung		4 (27)	4 (31)
	Apple iPhone		9 (60)	5 (38)
	LG		0 (0)	1 (8)
	Huawei		0 (0)	1 (8)
	Motorola		0 (0)	1 (8)
**Ability to use the functions of their smartphone^c^, n (%)**				
	Average		4 (27)	0 (0)
	Good		6 (40)	5 (38)
	Excellent		5 (33)	8 (62)
**Abilities to engage with and use other types of technology generally^c^, n (%)**				
	Poor		1 (7)	0 (0)
	Average		3 (20)	0 (0)
	Good		6 (40)	7 (54)
	Excellent		5 (33)	6 (46)
**Previously downloaded a mobile app to help increase their physical activity^d^, n (%)**				
	**Yes**		7 (47)	8 (62)
		Down Dog	2 (13)	0 (0)
		Fitbit	1 (7)	2 (13)
		Health	1 (7)	0 (0)
		Nike training club app	1 (7)	0 (0)
		Endomondo	1 (7)	0 (0)
		Sweat App	1 (7)	0 (0)
		Optimity	0 (0)	2 (15)
		Carrot	0 (0)	1 (8)
		Habitshare	0 (0)	1 (8)
		MyFitnessPal	0 (0)	1 (8)
		C25K	0 (0)	1 (8)
		ParticipACTION app	0 (0)	1 (8)
		7M workout	0 (0)	1 (8)
	**No**		8 (53)	5 (38)
**If you have previously downloaded an app to increase your physical activity, was it helpful?^d^, n (%)**				
	Yes		3 (20)	6 (46)
	No		4 (27)	2 (15)
**Physical activity behavior score, mean (SD)**				
	Moderate physical activity		—	54.29 (52.87)
	Vigorous physical activity		—	11.79 (20.90)
	MVPA^e^		—	66.07 (57.92)

^a^One participant did not complete the baseline questionnaire.

^b^Not applicable.

^c^Scale choices: terrible, poor, average, good, and excellent.

^d^Scale choices: yes and no.

^e^MVPA: moderate- to-vigorous physical activity.

#### Focus Group Findings

##### Overview

Refer to Table 2 for a comprehensive overview of the findings from the focus groups. Data saturation, where no new themes were revealed, was reached after 3 focus groups, which is in line with research stating that data saturation can be achieved with 3 to 5 focus groups [62].

**Table 2:** Multi-process action control (M-PAC) app usability focus group themes informed by the usability heuristics by Nielsen [54].

Heuristic theme	Positive findings	Negative findings and suggestions
Layout and information (including information on features)	The flow was nice and attractive and easy to navigate The 3 icons at the bottom were straightforward Liked the card format	Multiple levels of content for people who want more information The menu button should be reworded (does not take the user to a menu) Some people wanted the congratulations card before the reference card Trackers and self-reports should be in the same tab
Appropriateness of language	Language was appropriate for the general audience Lay terms that anyone could understand Fun theory	Language was for individuals who had higher physical literacy Once in a while, some words could have been different Some terms, such as self-efficacy, could be initially presented using a more widely known term Base screen says “activate account” instead of “create an account,” which is not intuitive
Backing out or undoing an action	Good that there were two options to go back—you could slide or also click Consensus that this was easy to do	Upon returning to a lesson from the home page, one needed to restart the lesson When they tracked a self-monitoring measure (blood pressure, PA^a^, or weight), it could not be edited or deleted Completed goals had a clickable “Completed” button, which made the goal “uncompleted” and took it back to the current tab
Consistency in language and features	Generally, people thought language and features were consistent	Modules versus lessons Icon differences in goals depending on Android or Apple phone (eg, “I get a little house a little running man and then something that looks like a golf a putting green flag”) Some things did not show up on iPad (long-term benefits with percentages)
Errors and mistakes	—^b^	Connection to Fitbit not working Some images did not load Some images were glitching (24-hour movement guideline) No way to reset the password For Android users, external links led to blank white pages Some images showed up too small
Forgetting acronyms	—	Sometimes an acronym was presented without defining it (MVPA^c^, CSEP^d^, FITT^e^, and PAR-Q^f^)
Efficiency of interface and navigation	Easy to swipe with 1 hand	Ability to connect to other PA-measure devices The swipe left button was too small Some tabs were swipe and click, while others were only click Desire to include the ability to link out to external web pages for references Each page should have a home page button
Visual design	People liked that similar images and illustrations were used People liked the color scheme People liked the representation among the images The design of the tabs was clean	Bold the FITT Too much text on some cards The external link placeholder image was too small and does not intuitively make people think it was a link Wanted more visual pop within the lessons Wanted the landscape format The check question button on the quiz answer card was not in an intuitive location On some pages, there was a lot of dead white space at the bottom One person wanted real images rather than the clip art More cards with less text When setting goals, the grey buttons were not intuitive and should be displayed in a different color for better clarity Program card could be better optimized (currently the title is cut off, and it flips to a sample page) Would like more color on some of the slides
Ease in diagnosing and resolving errors	—	Could not figure out password components (ie, how many letters or numbers were required) Needed to go through the entire set of help slides to find a solution The help button was hard to find
Help and documentation	—	Provided information on how to connect the Fitbit Help cards did not have a home button and could not be skipped The help button should be more accessible Ability to send error reports on specific cards, in the help tab, or at the end of modules Had to slide through the help slides individually Help icon could be changed
Additional features	—	Wanted to log other metrics, such as distance Want a progress bar The switch studies button should be a separate button Wanted a sound at the end of the lesson Desire to bookmark certain pages Desire for a toolbox for important questionnaires or favorite cards

^a^PA: physical activity.

^b^Not applicable.

^c^MVPA: moderate-to-vigorous physical activity.

^d^CSEP: Canadian Society for Exercise Physiology.

^e^FITT: frequency, intensity, time, type.

^f^PAR-Q: Physical Activity Readiness Questionnaire.

##### Theme 1: Layout and Information

Participants stated that overall the app was attractive and had straightforward icons. They also enjoyed that the app used cards to convey information, and they liked the flow when swiping between cards. However, participants disliked that the “Menu” button only allowed them to switch studies instead of lessons. Moreover, participants asked for multiple depths of content for people with various physical literacy levels.

##### Theme 2: Appropriateness of Language

Participants agreed that the app used lay terms to make the language appropriate and understandable for the general audience. Nevertheless, they suggested that a more PA-literate population might find the language too simple. In addition, the definition of some terms (eg, self-efficacy) could have been introduced earlier to enhance users’ understanding.

##### Theme 3: Backing Out or Undoing an Action

Participants liked that the app offered an easy way to navigate back from a page. For instance, they liked that the app had 2 options to return to the previous page (ie, by clicking or sliding). However, participants disliked that they needed to restart the lesson once they returned to the home page, could not edit or delete self-monitoring measures such as blood pressure and weight, and completed goals still had a clickable “completed” button, which uncompleted the goal.

##### Theme 4: Consistency in Language and Features

Generally, the participants agreed that the app’s language and features were consistent. However, participants reported inconsistency in wording and icons across different platforms.

##### Theme 5: Errors and Mistakes

Participants reported that the app could not connect to Fitbit. In addition, some images glitched (eg, showed up too small) or would not load. Participants also stated that they could not reset the password for the app. Another error was that external links led to blank pages for Android users.

##### Theme 6: Forgetting Acronyms

The focus group participants suggested that sometimes the lessons introduced acronyms (eg, MVPA, CSEP, FITT, and PAR-Q) without providing definitions.

##### Theme 7: Efficiency of the Interface and Navigation

The participants liked that they could easily swipe through the app when navigating. However, they reported that some tabs could only be clicked on, whereas others could be swiped through and clicked on. Participants also thought the swipe left button was too small, making clicking difficult.

##### Theme 8: Visual Design

The focus groups liked that the images and illustrations were coherent in style throughout the app. They also enjoyed the color scheme, visual representation, and the design of the tabs. However, participants did not like that key terms, such as FITT, were not bolded. They also disfavored that certain cards were text heavy.

##### Theme 9: Ease in Diagnosing and Resolving Errors

The participants reported that the app did not provide information about the password requirements when creating a password for log-in. They also disliked that the “help” button was hard to find, and they needed to go through the entire set of slides to find a solution to their problems.

##### Theme 10: Help and Documentation

Participants disliked that the app did not provide sufficient information on how to connect to Fitbit, the “Help” cards were not accessible (eg, the “Help” cards did not include a “Home” button and users could not skip to the card they wanted), and the lack of the ability to send error reports on specific cards.

##### Theme 11: Additional Suggestions on Features

Participants asked for features such as a progress bar in the lessons, a sound effect when finishing a lesson, a toolbox for important or favorite cards, a “home” button on each page, hyperlinks to reference articles, the ability to bookmark specific pages, and the option to input different PA metrics (eg, distance walked).

### Phase 2: Usability Pilot

#### Participants

Fourteen participants took part in phase 2 (usability pilot). One participant did not complete the baseline questionnaire due to unknown reason. Participants were mainly women (11/13, 85%) with a mean age of 41.38 (SD 12.92) years. Most (9/13, 69%) had at least a college or university degree, all (13/13, 100%) self-reported having above average abilities to use the functions of their smartphone, and most (8/13, 62%) had previously downloaded a mobile app to help increase their PA(eg, Fitbit, Optimity, Carrot, Habitshare, MyFitnessPal, C25K, ParticipACTION app, and 7M Workout). The most common brand of smartphone was Apple iPhone (5/13, 38%), followed by Samsung (4/13, 31%), Google Pixel (1/13, 8%), Hauwei (1/13, 8%), LG (1/13, 8%), and Motorola (1/13, 8%; Table 1). The participants reported on average 66.07 (SD 57.92) minutes of MVPA per week. None of the participants refused to participate or dropped out of the study.

#### Interview Findings

##### Overview

Refer to Multimedia Appendix 4 for a comprehensive overview of the findings from the interviews. A total of 14 interviews were conducted; however, only 13 interviews were recorded due to a malfunction of the recording instrument. Data saturation, where no new themes were revealed, was reached after 13 interviews, which is in line with research stating that data saturation can be achieved with 8 to 16 interviews [62].

##### Theme 1: Layout and Information

Of the 13 participants, 13 (100%) commented on the layout and information of the app, and everyone indicated something they liked (eg, straightforward layout and modules took a reasonable amount of time to complete). Of the 13 participants, 6 (46%) disliked the app’s layout and information due to inefficient content layout (eg, too many pages for a lesson), content and questions being too basic, and some content lacking references.

##### Theme 2: Appropriateness of Language

Of the 11 participants that commented on this theme, 7 (64%) acknowledged that the language used in the app was clear and easy to understand. However, of the 11 participants, 7 (64%) also commented on what they disliked regarding the app’s language choice, including the lessons not having descriptive titles. Some participants did not understand the meaning of the app’s name (M-PAC app) and thought it did not represent the app’s intent.

##### Theme 3: Backing Out or Undoing an Action

One (8%) participant commented on this theme and they mentioned that returning to the app’s home screen was inconvenient.

##### Theme 4: Consistency in Language and Features

Of the 6 participants that commented on this theme, 6 (100%) discussed the app’s consistency in language and features. All of them recognized something they disliked, including inconsistency in link descriptions (eg, some links had more details on why to click the link and some had no explanation), notification sounds appearing too frequently, and color labeling for the priority goals not following convention. By contrast, of the 6 participants, 2 (33%) liked that the app followed conventional color schemes (eg, active links were blue) and connected to conventional devices such as Fitbits.

##### Theme 5: Errors and Mistakes

Of the 11 participants that commented on this theme, 11 (100%) reported errors in the app. Some participants (4/11, 36%) stated that the app had problems connecting to Google Fit, and several links in the lessons did not work. Several users encountered general visual glitches, such as slow loading and pictures and data not being fully displayed on some devices. Other errors included the app being unresponsive, typos, problems with modifying weight and blood pressure information, step counts being inconsistent with other external trackers, and text not fitting on the screen.

##### Theme 6: Forgetting Acronyms

Of the 4 participants that commented on this theme, 3 (75%) did not understand what the app’s name (ie, Pathverse or M-PAC app) represents, and 1 (25%) participant forgot that PAR-Q stands for the Physical Activity Readiness Questionnaire.

##### Theme 7: Efficiency of the Interface and Navigation

Of the 13 participants that commented on this theme, 9 (69%) thought the app was overall well structured and easy to navigate. However, of the 13 participants, 7 (54%) discussed aspects they disliked about the app’s interface and navigation, such as the tracker feature being hard to find, not being able to go straight to the home screen after finishing a lesson, goals needing to be reentered daily, the tracking tab did not show dates for active minutes and daily goals, active minutes could not be entered for previous dates, and the progress of an unfinished lesson could not be saved.

##### Theme 8: Visual Design

Of the 13 participants that commented on this theme, 4 (31%) liked the app’s overall visual design, with comments stating that the app had visually pleasing graphics and engaging diagrams, and the color scheme helped user navigation (eg, blue active links turned purple when clicked). Of the 13 participants, 7 (54%) did not enjoy the app’s visual design for reasons such as the color scheme was not esthetic and lacked novelty (2/7, 29%), the graphic content did not include diverse body types (2/7, 29%), and the name and icon of the app were not attractive or meaningful to the users (3/7, 43%).

##### Theme 9: Ease in Diagnosing and Resolving Errors

Two (15%) participants commented on this theme and reported that no information was provided for the potential reason for errors (ie, no error messages or error codes).

##### Theme 10: Help and Documentation

One (8%) participant mentioned that the app did not provide information or warnings about upcoming updates.

##### Theme 11: Additional Feedback

Of the 13 participants that commented on this theme, 11 (85%) had a positive overall experience using the app. Many would likely use the app to improve their PA and recommend it to others. Of the 13 participants, 1 (8%) had a negative overall experience with the app. Some users stated that they would not likely use the app to improve their PA (3/13, 23%) or recommend it to others (4/13, 31%).

##### Theme 12: Additional Suggestions on Features

Most participants (11/13, 85%) suggested the features they would like to be included in the app, such as the ability to access new lessons overtime, take notes and make personal input during lessons, add comments to daily active minutes, and receive incentives on users’ PA progress.

#### Usability

A total of 13 participants filled out the MAUQ (Table 3). However, 1 participant did not fill out the MAUQ for unknown reasons. The mean total MAUQ score was 5.06 (SD 1.20), and the mean subscale scores were as follows: “ease of use” 5.36 (SD 1.27), “interface and satisfaction” 5.52 (SD 1.42), and “usefulness” 4.17 (SD 1.31).

**Table 3:** Mobile App Usability Questionnaire (MAUQ) scores of the pilot usability study (n=13).

Subscale	Score, mean (SD)
Ease of use	5.36 (1.27)
Interface and satisfaction	5.52 (1.42)
Usefulness	4.17 (1.31)
Total MAUQ score	5.06 (1.20)

## Discussion

### Overview

The purpose of this research was to gather user feedback on the first 2 prototypes of the M-PAC app to inform the design and development of an MVP. The ultimate aim of the M-PAC app is to promote sustained PA intention-behavior translation; consequently, it is informed by the M-PAC framework, which combines reflective and regulatory processes that assist individuals in translating PA intentions to behaviors with reflexive processes that contribute to PA maintenance [23,24,31]. We assessed the impressions of the first app prototype among a convenience sample of adults and subsequently evaluated the usability and acceptability of the second app prototype among a group of adults who intended to exercise but were not meeting the recommended PA guidelines. The M-PAC app design and development is informed by the IDEAS framework, which recommends an iterative phase of gathering user feedback and usability testing before feasibility and efficacy testing [38]. This step is important to ensure there is overall interest in the app, to identify potential impediments, and to collect suggestions for improvement and innovation.

### Principal Findings

Overall, the focus group and interview findings indicated that the M-PAC app was well received by target users and the usability scores were high. The findings also unveiled several suggestions related to usability that should be addressed before testing an MVP.

### Comparison With Prior Work

Participants thought the M-PAC app was easy to use and navigate, the layout was straightforward, and the lessons took a reasonable amount of time. Altogether, this feedback aligns with the “less-is-more” strategy, which has been proposed for effective human-technology interactions [63]. Enhancing the appeal and usability of the M-PAC app is crucial, especially considering that in 2022, the average retention rate for health and fitness apps dropped from 37% on the first day to just 9% by day 28 [13]. The most frequent reasons for abandoning mHealth apps are a lack of interest or declining motivation, preference for other apps, lack of desired features, lack of enjoyment (fun), and ease of use [64]. Consequently, the challenges with some of the features and navigation of the M-PAC app will need to be addressed, such as needing to reenter goals daily, inability to enter active minutes for previous dates, and inability to save lesson progress.

Several users reported difficulty with connecting to their PA tracking app. This is an important usability issue to address because users desire accurate and efficient tracking features [65]. Users also reported that the PA tracker feature was difficult to find and use (eg, activity minutes could not be entered for previous dates). Updating the PA tracking will need to be a priority because it facilitates self-monitoring, which is important for translating PA intentions into behaviors [10,11,66,67].

Another important characteristic to address is the app’s name. Several participants critiqued the name “M-PAC app,” stating it was not meaningful and did not represent the app’s intent. This is an interesting consideration that is specific to mHealth app behavioral interventions as it does not necessarily need to be addressed in face-to-face, educational leaflet, or website interventions. The app name is one of the first things that app users notice; therefore, it should be unique, easy to remember, and should clearly communicate the app’s functionality [68]. We began brainstorming potential replacements to the app name that are more understandable to a diverse audience and represent its intent to promote long-term PA behavior, and ultimately, we decided on the name “Physical Activity for Life (PAL) app.”

Participants voiced that they would like to continue accessing new lessons over time. This is supported by previous research stating that to maintain interest and encourage ongoing use of the app, users desire new and updated content [65]. Users desire tailored content, and to ensure they are receiving ongoing tailored content, a solution could be to implement the just-in-time adaptive intervention (JITAI) design. The JITAI design attempts to deliver support to the user “at the moment and in the context that the person needs it most and is most likely to be receptive” [69]. The M-PAC app development and design team is currently working on implementing a JITAI design.

The overall MAUQ scores from the usability pilot study were >5 for the total score, “ease of use,” and “interface satisfaction” subscales. Given that MAUQ scores of ≥5 indicate increasing levels of agreeableness, these scores suggest high usability [59]. The mean “usefulness” subscale score was <5, indicating moderate to low usability. This is not surprising considering participants only had access to the first 3 lessons of the app, while the MVP is estimated to have 13 lessons. This likely also reflects the earlier feedback indicating that participants wanted more content.

### Strengths and Limitations

Key strengths of the study are the sample demographics, particularly of the second study. Participants were aged, on average, 41.38 (SD 12.92) years, which is representative of millennials [70], who currently make up the most prevalent users of fitness apps [71]. Participants owned a range of different types of smartphone devices and were not currently meeting PA guidelines. A strength of the study design is that it used a mixed methods approach, including 2 types of qualitative feedback (eg, focus groups and interviews), as well as quantitative feedback in the form of the MAUQ. Mixed methods design offers unique and complementary insights into the multifaceted phenomena of mHealth usability [72]. Some limitations to the demographics are that the majority had a postsecondary degree and self-claimed to have good or excellent abilities to use the functions of their smartphone. Another limitation was that participants only had access to the first 3 lessons, out of the potential 13 lessons; however, the formatting will be the same for subsequent lessons, and the learnings from this study will be applied to those lessons as well. Because we are unaware of how the nature of regulatory and reflexive constructs may lend themselves to the current format, we plan to conduct further feasibility testing to more comprehensively understand whether the app is a viable means to deliver the complete intervention.

### Conclusions

Overall, the first 2 M-PAC app prototypes were usable and acceptable among the users. We will address the suggestions in the design and development of the MVP. Key upgrades we plan to implement include testing connections with the PA tracking feature, shortening lessons, coming up with a new app name, optimizing image loading, providing the ability to log activity minutes from previous days, providing the ability to add notes to PA logs, and enabling users to save progress in lessons. Once we have completed these modifications, we will conduct feasibility testing with the MVP.

## Appendix

Multimedia Appendix 1 Focus group interview guide.

Multimedia Appendix 2 Physical activity mobile health app informed by the multi-process action control (M-PAC) framework: lessons, mechanisms of action, and behavior change techniques.

Multimedia Appendix 3 Pilot study exit interview guide.

Multimedia Appendix 4 Physical activity mobile health app informed by the multi-process action control (M-PAC) framework: usability interview themes informed by the Nielsen’s usability heuristics by Nielsen [54].

## Abbreviations

BCT: behavior change technique
CONSORT: Consolidated Standards of Reporting Trials
COREQ: Consolidated Criteria for Reporting Qualitative Research
FITT: frequency, intensity, time, and type
IDEAS: integrate, design, assess, and share
JITAI: just-in-time adaptive intervention
MAUQ: Mobile App Usability Questionnaire
mHealth: mobile health
M-PAC: multi-process action control
MVP: minimum viable product
MVPA: moderate-to-vigorous intensity physical activity
PA: physical activity
SMD: standardized mean difference

## Data Availability

The datasets generated and analyzed during this study are not available because the participants only consented to providing the data to the research team of this study.
