# Supplementary material for: A Mobile Health App Informed by the Multi-Process Action Control Framework to Promote Physical Activity Among Inactive Adults: Iterative Usability Study
Source: JMIR Form Res. 2025 Apr 23;9:e59477. doi: 10.2196/59477 (PMC12059501; doi:10.2196/59477)
Supplement: Multimedia Appendix 1 [file formative_v9i1e59477_app1.docx]

## Appendix 1

### Focus Group Interview Guide

Thank you for your participation in our focus group study. The purpose of this study is to test the usability and satisfaction of a recently devolved physical activity promotion mobile phone application (app), called the M-PAC app. The data from these focus groups will be analyzed and shared with members of the research team to assist developers in the improvement of the M-PAC app.

From the completion of the workshop today we are curious to know how you have experienced the features of the app. Therefore, in today’s focus group I will ask questions about your experience.

Do you have any questions before we begin?

1. How did you find the layout and information of the app?
   1. If intuitive, what made the layout or information intuitive?
   2. If unintuitive, what made the layout or information unintuitive? What would be more helpful?
2. How did you find the appropriateness of the language that was used in the app?
   1. If understandable and clear, what contributed to the language being clear? Can you give an example?
   2. If difficult to understand or overly technical, where was language being used inappropriately? What would be more helpful?
3. How did you find the process of backing out of an action or undoing a process?
   1. Was it easy to undo or redo an action? What contributed to the ease/difficulty of this?
4. Did the language and features of the app seem consistent?
   1. If the language and features were consistent, what contributed to it feeling consistent?
   2. If the language and features were inconsistent, what could be changed to make it feel more consistent?
5. Did any errors or mistakes come up?
   1. Where did you notice the errors? How distracting were the errors? How much did the errors detract from your experience using the app?
6. Did you find that there were words or acronyms that were presented and then later on you forgot what they meant?
   1. What made these words or acronyms difficult to remember or recall?
7. How did you find the efficiency of the app user interface or navigating the app?
   1. If easy or intuitive, what made the app easy or intuitive to navigate?
   2. If difficult or unintuitive, what made the app difficult or unintuitive to navigate? What would be more helpful?
8. How did you find the content or visual design of the app?
   1. Did the app have an appealing design? If so, what contributed to the aesthetic appeal of the app?
   2. Was the app design too busy or unappealing? If so, what contributed to this? What would you modify to make it more appealing?
9. If you came across a problem or error, how easy was it to figure out a solution?
   1. If easy, what contributed to making the error or problem easy to address?
   2. If difficult, what contributed to making the error or problem confusing or difficult to address?
   3. At times when you needed guidance or assistance in the app, how useful did you find the information provided by the app to help?
   4. If the help provided by the app was adequate, what contributed to the information being useful?
   5. If the help provided by the app was inadequate or not useful, what would make the information more useful?
