# Supplementary material for: A Mobile Health App Informed by the Multi-Process Action Control Framework to Promote Physical Activity Among Inactive Adults: Iterative Usability Study
Source: JMIR Form Res. 2025 Apr 23;9:e59477. doi: 10.2196/59477 (PMC12059501; doi:10.2196/59477)
Supplement: Multimedia Appendix 2 [file formative_v9i1e59477_app2.docx]

## Multimedia Appendix 2

**Supplementary Table 1. Multi-process action control (M-PAC) app lessons, mechanisms of action, and behavior change techniques (BCTs).**

| M-PAC app module | M-PAC constructs | BCTTv1^a^ | BCT label and ID | Use example in the M-PAC app | Educational material |
| --- | --- | --- | --- | --- | --- |
| Lesson 0: PAL^b^ app tutorial and introduction | N/A^c^ | —^d^ | — | Educational cards | Layers of M-PAC  Why the M-PAC app |
| Lesson 1: what is PA^e^ | Instrumental attitude | Information about health consequences (5.1)  Behavioral practice or rehearsal (8.1) | Inform about health consequences BCT (BCIO^f^:007063)  Practice behavior BCT (BCIO:007094) | Educational cards  3 quiz cards  1 activity card | 24-hour movement guidelines for adults (aged 18-64 years) and for older adults (aged >65 years)  Get Active Questionnaire |
| Lesson 2: health benefits of PA | Instrumental attitude | Information about health consequences (5.1)  Behavioral practice or rehearsal (8.1) | Inform about the health consequences of BCT (BCIO:007063)  Practice behavior BCT (BCIO:007094) | Educational content  YouTube video from Dr Mike Evans on the health benefits of PA  3 quiz cards  1 reflection card  1 activity card | Short- and long-term physical health and mental benefits of PA  Productivity and self-efficacy benefits of PA |
| Lesson 3: self-efficacy | Perceived capability | Goal setting (1.1)  Instruction on how to perform behavior (4.1)  Self-monitoring (2.3)  Demonstration of the behavior (6.1)  Behavioral practice or rehearsal (8.1)  Graded tasks (8.7)  Distraction (12.4) | Goal setting BCT (BCIO:007002)  Instruct how to perform a BCT (BCIO:007058)  Self-monitoring (BCIO:007024)  Demonstrate the BCT (BCIO:007055)  Practice BCT (BCIO:007094)  Set graded tasks BCT (BCIO:007100) | Educational content  3 quiz cards  1 reflection card  1 activity card | Define self-efficacy  How to build self-efficacy  FITT^g^  Progression principle |
| Lesson 4: learning about your emotions | Affective attitude  Behavioral regulation  Emotion regulation | Information about emotional consequences (5.6)  Monitoring of emotional consequences (5.4)  Remove aversive stimulus (7.5)  Adding objects to the environment (12.5)  Behavioral practice and rehearsal (8.1)  Biofeedback (2.6)  Self-incentive (10.7)  Reduce negative emotions (11.2)  Self-talk (15.4) | Inform about the emotional consequences of BCT (BCIO:007065)  Monitor the emotional consequences of BCT (BCIO:007066)  Provide biofeedback on BCT (BCIO:007026)  Promise positive consequences for behavior (BCIO:007202)  Advise on how to reduce negative emotions BCT (BCIO:050344)  Prompt self-talk BCT (BCIO:007140)  Remove aversive stimulus BCT (BCIO:050331)  Add objects to the environment BCT (BCIO:007156)  Practice behavior BCT (BCIO:007094) | Educational content  2 quiz cards  1 reflection card  3 activity cards  Images to portray ACT^h^  Video from Headspace about mindfulness  Video from Headspace on a breathing exercise  Video about how Michael Phelps uses visualization to succeed | How PA enjoyment reflects on participation  How to foster PA enjoyment  Describe emotion regulation  Describe the difference between affect, emotions, and moods, and what influences affect  How PA influences affect  ACT  Psychological flexibility  Mindfulness  Self-compassion  Visualization  Mental toughness |
| Lesson 5: social support | Perceived opportunity | Goal setting (1.1)  Self-monitoring (2.3)  Social support (practical; 3.2)  Social support (emotional; 3.3)  Behavioral practice and rehearsal (8.1) | Goal setting BCT (BCIO:007004)  Self-monitoring (BCIO:007024)  Advise to seek emotional support (BCIO:007031)  Arrange emotional support BCT (BCIO:007036)  Deliver emotional support BCT (BCIO:007041)  Practice behavior BCT (BCIO:007094) | Educational content  1 quiz card  1 reflection card  1 activity card | Creation of social opportunities  4 types of social support |
| Lesson 6: building PA opportunities | Perceived opportunity | Restructuring the physical environment (12.1)  Behavioral practice and rehearsal (8.1) | Restructure the physical environment (BCIO:007156)  Practice behavior BCT (BCIO:007094) | Educational content  1 quiz card  1 reflection card  1 activity card | Build environmental opportunities  Grab-and-go activities |
| Lesson 7: goal setting and planning | Behavioral regulation | Goal setting (1.1)  Problem-solving (1.2)  Action planning (1.4)  Prompt and cues (7.1)  Behavioral contract (1.8)  Behavioral practice and rehearsal (8.1) | Goal setting BCT (BCIO:007004)  Set behavior goal BCT (BCIO:007003)  Create behavioral contract BCT (BCIO:007014)  Goal strategizing BCT (BCIO:007008)  Action planning BCT (BCIO:007010)  Prompt intended action BCT (BCIO:007080)  Cue BCT (BCIO:007081)  Practice behavior BCT (BCIO:007094) | Educational content  3 quiz cards  1 reflection card  1 activity card | Describe what SMART^i^ goals are and why they are beneficial  Flexible goal setting  Describe action planning and its different subcomponents  How to use coping planning  “If...Then...” statements |
| Lesson 8: self-monitoring | Behavioral regulation | Self-monitoring (2.3)  Behavioral practice and rehearsal (8.1) | Self-monitoring (BCIO:007024)  Practice behavior BCT (BCIO:007094) | Educational content  1 quiz card  1 reflection card  1 activity card | Describe self-monitoring  Understand the importance of self-monitoring  Recognize different ways to self-monitor |
| Lesson 9: PA habit | Habit | Prompt and cues (7.1)  Associative learning (7.8)  Behavioral practice and rehearsal (8.1)  Habit formation (8.3) | Prompt intended action BCT (BCIO:007080)  Cue BCT (BCIO:007081)  Context-specific repetition of behavior BCT (BCIO:007096)  Habit learning (BCIO:006309)  Practice behavior BCT (BCIO:007094) | Educational content  3 quiz cards  1 reflection card  1 activity card  Video about habits | Relation of habit to PA  Habit formation (repetition, script, and environmental cues) |
| Lesson 10: identity | Identity | Social comparison (6.2)  Incompatible beliefs (13.3)  Framing and reframing (13.2)  Valued self-identity (13.4)  Identity associated with changed behavior (13.5)  Behavioral practice and rehearsal (8.1) | Prompt social comparison BCT (BCIO:007073)  Draw attention to incompatible beliefs BCT (BCIO:007057)  Reframe past behavior BCT (BCIO:007056)  Affirm valued self-identity BCT (BCIO:007159)  Practice behavior BCT (BCIO:007094) | Educational content  Image of key contributors to a strong exercise identity  2 quiz cards  1 reflection card  1 activity card | The idea of exercise identity  Ways to increase exercise identity (commitment, enjoyment, social comparison, and passion) |

^a^BCTTv1: behavioral change technique taxonomy version 1.

^b^PAL: Physical Activity for Life.

^c^N/A: not applicable.

^d^Not available.

^e^PA: physical activity.

^f^BCIO: Behavior Change Intervention Ontology.

^g^FITT: Frequency Intensity Time Type.

^h^ACT: Acceptance and Commitment Therapy.

^i^SMART: specific, measurable, achievable, relevant, and time-bound
