# Supplementary material for: A Mobile Health App Informed by the Multi-Process Action Control Framework to Promote Physical Activity Among Inactive Adults: Iterative Usability Study
Source: JMIR Form Res. 2025 Apr 23;9:e59477. doi: 10.2196/59477 (PMC12059501; doi:10.2196/59477)
Supplement: Multimedia Appendix 3 [file formative_v9i1e59477_app3.docx]

## Multimedia Appendix 3

### Pilot Study Exit Interview Guide

Thank you for your participation in our study. The purpose of this study is to test the usability and satisfaction of a recently developed physical activity promotion mobile phone application (app), called the M-PAC app. The data this interview will be analyzed and shared with members of the research team to assist developers in the improvement of the M-PAC app.

Do you have any questions before we begin?

Do you consent to participate in the interview and that it is being recorded (audio)?

1. How was your overall experience with the M-PAC app?
2. Can you tell me what you liked best about the M-PAC app?
   1. Were there any features in particular?
   2. What about the content and/or layout?
3. Can you tell me what you liked least about the M-PAC app?
   1. Were there any features in particular?
   2. What about the content and/or layout?
4. Can you tell me about whether or not you would use such an app to learn how to better manage your physical activity? Why?
5. Can you tell me about whether or not you think others would be interested in using this app to learn how to better manage their physical activity? Why?
6. If you could change anything about the M-PAC app, what would you change?
   1. Are there any features you would add or remove? Why?
7. Is there anything else you would like to tell us about the M-PAC app?
