# Supplementary material for: A Mobile Health App Informed by the Multi-Process Action Control Framework to Promote Physical Activity Among Inactive Adults: Iterative Usability Study
Source: JMIR Form Res. 2025 Apr 23;9:e59477. doi: 10.2196/59477 (PMC12059501; doi:10.2196/59477)
Supplement: Multimedia Appendix 4 [file formative_v9i1e59477_app4.docx]

## Multimedia Appendix 4

**Supplementary Table 2. M-PAC app usability interview themes informed by Nielsen’s usability heuristics.**

| Heuristic Theme | Liked / Dislike / Suggestions (Number of respondents) | Component (Number of respondents) | Example quotes |
| --- | --- | --- | --- |
| Layout and information (13 respondents) | Disliked (6/13) | The content was too basic (4) | “I would probably read them just to see if there was anything in there for me to learn. But I found this pretty basic, so, you know.” [3]  “Um, I reviewed the content a couple of times. I feel like I've seen some of it before through other apps, so maybe I didn't, I didn't connect with it as much.” [5] |
|  |  | Content layout was inefficient (1) | “I can imagine this being, like, all condensed into, like, a five-minute video and not necessarily, like, swiping through all of these cards to read, or I can imagine it just being on one single webpage and just, like, read out that way. That's the main thing I felt is that, because there are so many pages, whenever you flip a page, you feel like there's some new content, but it's actually a lot of just continuation of, like, the same topics.” [8] |
|  |  | Missing references (1) | “In the introduction lesson zero, on slide 11 of 15, there was mention of smart goals and overcoming barriers, and I didn't really find specific references to those two things. Maybe those are later lessons. Um, so, where that content is was one of my questions.” [1] |
|  |  | Questions were too simple (1) | “I think I would change the, the question-and-answer part. I think the questions were in some cases maybe a little bit lacking, or the answer was so obvious that it was like, ‘ugh.’ *Chuckles*” [1] |
|  | Liked (13/13) | Challenge questions were helpful and had reasonable difficulty (6) | “It made you have to think back to all the things that you've read during the different modules.” [9]  “Um, they weren't too hard, but they also weren't too easy. Like, for some of them, I had to think about it. So, I think it was perfect, honestly.” [10] |
|  |  | Challenge questions were spaced throughout the modules (1) | “Yeah, having the challenge cards were, that was good, placed throughout the modules rather than one big one at the end. So that was a good feature.” [3] |
|  |  | Enjoyable and well-expressed content (10) | “I think the health benefits section I found the most, um, well expressed, I think. *Pause* And I enjoyed that section the most. It's nice to hear what good will come of something.” [1]  “So that thing I liked later in the lessons when it mentioned even just a few minutes of exercise per day can have a positive effect on health. That actually rang more true to me and felt more achievable.” [2] |
|  |  | Information was introduced gradually (1) | “It starts off with the small lessons, like bite-size pieces and step-by-step, and it doesn't even start off with exercising. It's really understanding why and all that. So, I really like that and step by step in that way.” [10] |
|  |  | Modules took a reasonable time to complete (7) | “I found they were easy to just get through it, so I wasn't sitting there too long trying to get through each one.” [9] |
|  |  | Informative content (4) | “Oh, that was, that was exciting. Informative. Informative and interactive.” [6]  “It was very informative, easy to navigate, easy to use. It was a positive experience.” [7] |
|  |  | Page numbers indicated current progress (1) | “And I appreciated that it had the screen, what screen number was at the bottom so that I knew how many more screens I had to go, where I was in the process for that module.” [3] |
|  |  | References provided helpful information (1) | “I would keep the references in there. *Chuckles* I don’t know if that's because for people like myself, not everybody, like, I know my husband said, ‘I would not read the references.’ But for people like myself that love more information and accurate data, I found that, oh, I found that so interesting.” [2] |
|  |  | Straightforward layout (5) | “I found the layout was simple. It was easy for everyone.” [9]  “What I like the best is the ergonomics, the structure of the application, and the quality of the lessons.” [12] |
|  |  | Easy access information (1) | “So, I think being able to track or maybe having an app that gives guidance or advice on different things to try to get the motivation I think would be helpful. And I think that would, and having it on your phone is pretty easy cuz we all take our phones everywhere we go anyway. So, it's always there.” [9] |
|  |  | Had the option to input users' body measurements (1) | “And I liked being able to input, like, your, your weight and your measurements and all of that if you wanted to.” [10] |
|  |  | The app offered multiple ways to gain information (1) | “I think it was perfect, honestly. And I like the mix of, like, some questions here and if I wanted more information, there were links, or if I wanted video, I could watch the video, cause everyone learns differently and I liked having the option and the, the variation and those little questions were able to, like, get me back to focus on what was going on, even if it was very short.” [10] |
|  |  | The app had a tracking function (1) | “I did like that I've been able to, was able to track how many, like, how long I've walked.” [9] |
|  |  | The app showed users’ progression towards lesson completion (1) | “Oh, it's the, the completed, each time completed, you know, how many, how many lessons you did, you have like a tracking, you have a good tracking of, of where you are, your level of lessons completed. Everything is tracked.” [12] |
|  | Suggestions (10/13) | Ability to access multiple lessons per day (1) | “Um, maybe the option to access more than one lesson per day if you wanted to. So, on some days, some people are really motivated, and on some days, you might not be. But maybe you're trying to build a habit, so I'm gonna sign in every day and do one lesson a day. But maybe you're like, ‘Oh, I actually, I wanna do a bit more today. I wanna learn more; I'm into it.’ And then you could have the option.” [10] |
|  |  | Add overview and objectives for each module (1) | “So, kind of the top, I personally prefer learning from like a top-down approach. I like how there was the objective in the beginning, like a summary of what you might be learning in this lesson, but I felt that it would be even nicer to get to see an overview of the topics that would've been going through because I felt that, ‘oh, I know going in there's an objective and stuff,’ but as I keep scrolling it, I feel like more and more ideas came up and, and it got pretty hard for me to capture the, the main idea of what the lesson was trying to deliver.” [8] |
|  |  | Add various forms of content to keep users’ attention (2) | “So, it was very text heavy. So, I thought that maybe visual aids and videos could help. And I, I don't always like reading long text, and when I see like, a long list of, uh, of words, sometimes I go, ‘oh, so much to read.’” [7] |
|  |  | Enable users to go back to previous dates to input data (1) | “I think being able to go back if you missed a day to put the numbers in is really important. Um, and that's sort of like a, *Pause* it's very rare to not be able to do that on app, to be honest… I see the dates and I can go, ‘Oh, I forgot to enter on that date. I want to be able to hit that date and be able to put my information in.” [2] |
|  |  | Include instructions for users to properly perform activities (1) | “Mm, I guess with the exercise, maybe more detailed, um, kind of types of exercise and how to perform it. I don't know. And that's just what I was thinking, like visual kind of pictures and like how to do like for example, like a squat, how to position yourself to prevent injuries or something like that.” [13] |
|  |  | Introduce new content over time (4) | “For content? Like, I'm not sure how it will roll out later, but if I had, like, extra content each week instead of like, just all at once, that would be much better for me to learn something new. Yeah.” [5]  “The added value for me was the content, the lessons. But if those lessons don't update, I may not come back to the app.” [7] |
|  |  | Notice the users about quizzes in advance (1) | “‘Oh no. Well, I, I had, did I remember everything I read in those pages?’ So, I think it would be nice at the beginning to say there will be little, short quizzes.” [2] |
|  |  | Let users see the summary of their quiz answers (1) | “So, that's one thing that could happen even if it wasn't considered correct or incorrect to have certain answers. It would just be like cool to get to see what people fill out throughout each of these sections.” [8] |
|  |  | More information on individual slides instead of having multiple slides (2) | “Cause there's a lot of slides with relatively few words. So, I might, if I were building it, have more words and fewer slides or just more words. Maybe that's my preference for reading more.” [1] |
|  |  | More lessons with less content in individual lessons (4) | “Well, the thing is, you know, like, there's a couple of sessions, the slides, you know, the slides that you have to go to, one out of 28 or… so, if, you know, if instead of 28, 29, wondering if you can further subdivide it into categories, instead of just 28, you keep on sliding it, you know, if you can just divide it into five, six slides.” [6] |
|  |  | Provide descriptions about the reference links (1) | “Provide, my assumption was it would provide more information that was relevant to a specific part of the course. Either to a specific lesson or a specific topic. But I wasn't so much interested in like, ‘this is the research material that goes with lesson three.’ I was more interested in ‘this is the research material that goes with the concept of reducing your blood pressure’ or whatever it was, so…” [1] |
|  |  | Provide more explanations on important concepts (1) | “Um, slide 13 of 27 about the improved quality of life, I thought was a really, really important concept to get across and that is another one that might be expanded upon. And 15 of 27 I thought needed more expansion as well.” [1] |
|  |  | Show dates under the tracking tab to show users’ progress (1) | “So, when I, yeah, I want, I wanna see a date at the top when I go to hit my daily goal and active minutes and steps, I'd like to see a date there.” [2] |
|  |  | Some content can be more concise and condensed (1) | “Because I don't think it's, yeah, there are, like, too many cards, but there are too many cards because the, like, the same info as we presented, like, broken down into more cards. So, I think maybe for some of the lessons, the content, it may have been appropriate to be in the single lesson rather than two.” [8] |
| Appropriateness of language (11 respondents) | Disliked (7/11) | Content titles were not descriptive (1) | “Lesson one, lesson two. You know, these appear very like a kind of a generic, you know… so, if something like lesson one, ‘how to be more active,’… something like that, you know... information that's kind of up-to-date, information regarding our physical activity.” [6] |
|  |  | Jargon used with inadequate explanations (3) | “It's describing self-efficacy, and it just, see, I understand the concept of efficacy, but it seemed a confusing way to get it crossed.”  [1] |
|  |  | Language did not follow conventions familiar to users (2) | “Because it's, it's like, ‘oh, this isn't a local one, this is somebody else’s…’ Yeah. It made me go, ‘I'm old school, so, you need to spell behaviour correctly.’” [3]  “The last thing I wrote here was that weight was only in pounds. I could only put my weight in pounds. So, maybe have some kilograms option.” [7] |
|  |  | Name of the app was not meaningful to users (3) | “Um, I think it is the name of the app or is it the, is that the name of the program that…” [4] |
|  |  | Voicing of the content was too academic (1) | “Slide nine of 27 was another sort of rubs me the wrong way thing. It just started with ‘research shows,’ which is academic-speak, as opposed to exercise app, speak. *Chuckles*” [1] |
|  | Liked (7/11) | Clear language usage (7) | “I think the health benefits section I found the most, um, well expressed, I think. *Pause* And I enjoyed that section the most.” [1]  “It was easy to read. It was logical, it was fairly basic.” [3] |
| Backing out or undoing action (1 respondent) | Disliked (1/1) | The app did not automatically return to the home screen when a lesson is completed (1) | “I think the only thing is when you are finished, I wish it would go back to the home screen, instead of having to click the home button before it went back to the home screen. So it was just between the lesson that it was, it took a bit.” [11] |
| Consistency in language and features (6 respondents) | Disliked (6/6) | Inconsistency in link descriptions (1) | “Some of the slides in that section had more details explaining why to click the link and some had less.” [1] |
|  |  | Inconsistency in app navigation (5) | “So, it's more, I think at this age we are more intuitively wanting to, you know, swipe left, swipe right or like click right, click left to have something happen.” [8]  "Yeah, just like pressing like, the next button instead of having to press home and then click on the next module manually.” [7] |
|  |  | Notification sounds came up too frequently (1) | “The buzz. Yeah. It started to get a little annoying through 20 sets of cards.” [8] |
|  |  | Colours for the goals did not follow conventional themes (1) | “So, yeah, like for goals I, yeah, like they’re red, blue, green goals, but green and blue, like, I think blue was like a low priority goal and I always kept clicking on like, I, I feel like blue should be a medium priority goal. Green should be low.” [5] |
|  |  | Zoom didn’t function as expected (1) | “And when I press zoom, it only zoomed in on the picture, but not the text…you can’t zoom manually with your fingers at all.” [7] |
|  |  | Buttons were too small on some devices (1) | “And also, the next button is kind of small for, even for my phone, my phone’s like not the smaller phone, but it’ll be kind of hard to press on it if it’s like super, super small.” [8] |
|  |  | Forget where things are (1) | “I did. Uh, I, yeah, I broke down the goal and then, you know, I had to go back to find it.” [4] |
|  |  | Some data were not fully displayed (1) | “Cause, like, I find with some devices you can see it, but you can’t fully see, like, the different data. Like, how like these have the graphs if you like to show went up or down.” [9] |
|  |  | Unclear intent of the app (1) | “Um, so, I think I’m getting confused between it being a habit app or physical activity app.” [4] |
|  |  | Goals needed to be entered daily (1) | “Like, every day I had to enter the goal, and I found that very frustrating because it was like, I, I already entered…” [2] |
|  | Liked (2/6) | Colours followed conventional schemes (1) | “You know, blue was an active link. So, the conventions of color were very simple and recognizable.” [1] |
|  |  | Connects to conventional devices (1) | “…It has the steps and you can connect it to your different devices, whether it's a Fitbit or any, and everything like that. So, if it has, I think everything that connects to the different devices, we, most people already have.” [9] |
| Errors and mistakes (11 respondents) |  | App was unresponsive (1) | “I don't know why this app is not working anymore, interestingly.” [6] |
|  |  | General glitches (3) | “It was okay. It was a little frustrating in that there were glitches, it was a lot of glitches.” [2]  “Um, I did come across, a couple of glitches, minor glitches, mostly just visual, but otherwise, it was good.” [3] |
|  |  | The tracker had issues connecting to Google Fit (5) | “The tracker is a good idea. It didn't work that well for me. My Google Fit doesn't work that well. It doesn't interact with my various body monitoring devices. So that was kind of, to use an acceptable term, clunky.” [1]  “But I noticed when, you know, how you were speaking about how you could connect your steps with, like, your iPhone? I dunno if maybe it's cause the app is new, but I wouldn't find, for one thing, it wouldn't let me connect it to Google Fit.” [9]  “I liked, or I would've liked being able to connect my, I tried connecting Google Fit and it didn't work.” [10] |
|  |  | Pictures loaded too slowly (3) | “So, some, quite a few slides, actually, I had read all the text content before the graphic got there, so I was often scrolling through before I got to appreciate the graphic. So, faster loading would be good.” [1]  “Oh, there were a couple of, I noticed that, that there had, there was one page where they, the visual, the little picture wasn't on it, but it's on now. I checked this morning.” [3] |
|  |  | Several links did not work (4) | “Yes, some of the links didn't work, which may have been my end because of my slow internet connection. Sometimes things just don't load properly if they're a significant size.” [1]  “Oh. A big one is links didn't open in any of the sessions.” [7] |
|  |  | Typos (1) | “Other things, there was, there was some typos. But nothing too big. A few extra dots here and there.” [7] |
|  |  | Unable to enter or change weight and blood pressure information (2) | “Um, also, you could not, let's see, you could not enter the weight, the blood pressure.” [2]  “But the other issue that I had in my notes was that for earlier, I couldn't change, I wasn't able to change the weight part, but now I can.” [9] |
|  |  | Step counts were inconsistent with other external trackers (1) | “The steps, it was synced to Apple Health, and it was so much lower than what my actual steps were. Hmm. How do I know that? Cuz my Fitbit showed, my Fitbit was like triple the amount.” [2] |
|  |  | Text did not fit on screen (1) | “Mainly the answers after you've taken the quiz. And it says, ‘yes, you know this, you got the right answer.’ And then at the bottom, the last line didn't quite show. It's not terribly relevant. I get the rest of it, but yeah, I'm sure as someone who's written the, the screens want to know that you can't quite see the bottom. Yeah.” [3] |
| Forgetting acronyms (4 respondents) |  | Forgetting the PARQ acronym (1) | “The link, yeah. Yeah, the Q pack or…PARQ, that's what I'm, that's what I'm, yeah. PARQ link did not work.” [1] |
|  |  | Users did not understand what the app’s name stands for (3) | “Um, I, I don't like the, the name, I don't understand what the name is.” [3]  “Pathverse, like, Pathverse, what does this term mean?” [6] |
| Efficiency of interface and navigation (13 respondents) | Disliked (7/13) | Confusion on how to access the tracker feature (1) | “So now what, you know, it's like, where do I go to get my tracker?” [3] |
|  |  | Must push ‘home’ to go to the home screen after finishing a lesson instead of going straight to home (1) | “I think the only thing is when you finished, I wish it would go back to the home screen, instead of having to click the home button before it went back to the home screen. So it was just between the lesson that it was, it took a bit.” [11] |
|  |  | Had to re-enter the goals everyday (2) | “Same on goals. It has ‘current’ and ‘completed’. It was weird on the goals, it would say ‘daily’, but it wouldn't show up next day and I have to re-enter the goal.” [2]  “Being able, oh yeah, I said that like I, I just said to put in goals for multiple days, like, so that I actually have my, the goals stay there instead of having to, um, put in new ones, that would be handy too.” [5] |
|  |  | No dates for goals under the tracking tab (1) | “Like, when you go to trackers, it says active minutes and daily goal, but there's no date anywhere.” [2] |
|  |  | Not able to enter active minutes for previous dates (2) | “I think being able to go back if you missed a day to put the numbers in is really important. Um, and that's sort of like a, *Pause* it's very rare to not be able to do that on app, to be honest.” [2] |
|  |  | Not able to move directly to the next module after finishing a module (1) | “Oh, something I said I thought was annoying is that when you finish a module, you, it, would it be nice if I could just do next and move on to the next module, but I couldn't, I had to go to home and then click on the, it's, it's really petty. It's really petty.” [7] |
|  |  | Same content was broken up into multiple pages (1) | “So, for instance, sometimes I realize some of the content was even broken up. So, the first page might say something like, the first point is this and … and then you have to swipe to get to the second point, for instance. I feel that it just really like dragged down over a while.” [8] |
|  |  | The app did not save the progress if the lesson was unfinished (2) | “In each lesson, you had to do the full lesson in one sitting because if you didn't, when you came back, you had to start at the beginning.” [2]  “I didn't really try, but I don't know if you go back after you've started the lesson, if it marks it as half complete and you have to go back to the beginning or not. Cuz I kind of went through all the slides.” [11] |
|  |  | The data input option (e.g., inputing weight) was not user-friendly (1) | “The, yeah, the input stuff. It was, you know, manually, I, you know, like before you said you might attach it to something, but to try to put in your, you know, your weight and your, what was the other thing? The, your heart rate and stuff? It was, you know, sort of not tedious, but a little bit more manual, you know, and I don't feel like we're at that point anymore. It's like, like it should just be able to know what you're doing.” [4] |
|  |  | The notification function was tricky to turn on (1) | “*Laughs* But I would love, I couldn't really figure out how to get notifications to work and so like, a daily reminder to get something done would help me a lot if that's not already there.” [5] |
|  |  | Inconsistent ways of swiping in the app (1) | “It's, I'm trying to think of like the, the, sometimes it's so confusing when you have different ways of, of swiping or like, you know, like these little files that open up here and then open up here, open up here.” [4] |
|  | Liked (9/13) | Clear navigation through the app (9) | “It was good. It was easy to use and follow… Um, it was easy to move from screen to screen.” [3]  “Um, it was structured easily, so I knew where I needed to go.” [7] |
|  | Suggestions (1/13) | Add a navigation feature that allows users to navigate from page to page (1) | “Oh, and also going back to features, it'll be cool to have a navigation feature because I can't navigate from page, like, from page one all the way to page 14 without like having to…Yeah. Because I would love to have that right now if I could.” [8] |
|  |  | Create an easily accessible exit for the app (1) | “…but it'll be cool if you want to like exit out of the app to have a function where you drag the, like the leftest part of the screen all the way and then it would make the entire content, like, go back to the main menu rather than clicking.” [8] |
| Visual Design (13 respondents) | Disliked (7/13) | Colour scheme was not aesthetic and lacked novelty (2) | “All right. Yes. This is, yes, this, for example, I would, I would like, I would like more, more aesthetic. I find it. Yes. I find it too cold. You know, it's too clinical.” [12]  “Uh, in some ways, probably kinda the same thing. It was very conventional in terms of the color scheme and so that was both good in terms of its ease and comprehensibility, but maybe a little less engaging than something with a little bit more novelty or a bit more unique look and feel to it.” [1] |
|  |  | Graphic content did not include diverse body types (2) | “I think your characters are okay, but they're all so thin. I think I would have more realistic-looking bodies. *Pause* I know that seems really silly.” [2]  “I dunno if they were so inclusive though. Like, if I have to be super critical, I feel like, yeah, there was, you know, everybody looked able-bodied and you know, I mean there was, you know, I felt like there was a range of, of skin shades, but there wasn't a range of differences in some of those other things like age and, um, yeah, abilities, you know?” [4] |
|  |  | Some pictures were too large that they interfered with the text (1) | “I think some of the pictures were, I mean like they were really, really big, so you kind of had to scroll to get to the actual wording. Like, if the pictures were a little bit smaller or like the wording was on top and the pictures at the bottom or something. Cuz sometimes the picture didn't load quite right. And so, the wording would be just like bouncing as the pictures are loading. *Chuckles*” [11] |
|  |  | The correct answers to the quiz questions were not highlighted (1) | “Yeah. Oh, after getting question right in the text, the correct answer was not highlighted. So, like, I, I, so, so when I, when I answered the quiz, so I got the question right. And then I went back to the, the, the lesson and then the right answer wasn't highlighted. I don't know why I expected it to be highlighted as if, ‘oh, here, that was the right answer, but it didn't.’” [7] |
|  |  | The name and icon of the app were not attractive (3) | “Um, I don't know what would attract them if they didn't know about it already. Like, I've got a, I have a friend that I would probably recommend she try because she's not getting enough exercise and she's not, she needs some more motivation to get at least moving. But how she would, you know, I don't think she would be attracted to it, the name doesn't attract me. I don't know how you would visually display it for someone to be drawn to it kind of thing.” [3]  “No, like you said, like if I was gonna go to *Inaudible* and download the app, like the little icon or the, and I mean it's, it's kinda like the cover of the book, right?” [4] |
|  | Liked (4/13) | The color scheme helped navigation (1) | “I think you've actually got the color choices, that blue, in, in people's minds that like blue has become the active link. You know, you click the blue. If it turned to purple after it was used, that would be even more obvious, that convention of blue purple. But really, because you want people to be able to go back to lessons and leaving it blue is also a pretty valid choice. So Okay. And the red, yellow, green bits, that's also a very obvious convention and yeah, so I think anyone not familiar with apps will find those colors, make their path much easier.” [1] |
|  |  | Engaging diagrams (1) | “I love that the diagrams are really engaging.” [8] |
|  |  | Visually pleasing graphics (3) | “And I think that helped it be a more positive experience. It's also put in positive ways the graphics are cheery. It in general has a positive feel.” [1]  “The pictures were cute. The colored pictures were, yeah, appealing.” [4] |
|  | Suggestions (6/13) | Add congratulatory animations once a goal is reached (1) | “Um, whenever I click on a goal to say that I have achieved that goal, like, if it would do something interesting, that would be great. Like confetti or something.” [5] |
|  |  | Introduce more visual aids (4) | “If they just, if there was like a little animation that kept it like, this is, this is the information here. It's not just reading it, um, reading's not my first go-to. I'm visual, but, yeah, just a thought.” [4]  “So, it was very text heavy. So, I thought that maybe visual aids and, and videos could, could help. And I, I, I don't always like reading long text and when I see like, a long list of, uh, of words sometimes I go, ‘oh, so much to read.’” [7] |
|  |  | The visuals should be more accessible for people with disabilities (1) | “Something else that I thought was an issue, um, was that I was wondering if, if you could do something that can be more, make the app more accessible for people with disabilities. So, larger font or text speech feature. Like, I wasn't able to increase the text if I wanted to.” [7] |
| Ease in diagnosing and resolving errors (2 respondents) | Disliked (2/2) | No information provided for the potential reason of errors (2) | “But I got a white screen, I didn't get an error message or an error code.” [1]  “So, I didn't know if it was supposed to go somewhere or just because it was a test. It's not there yet.” [3] |
| Help and documentation (1 respondent) | Disliked (1/1) | The app did not provide information about upcoming updates (1) | “And yes, it's to change, but to, to, because for example, me, I was not, you have to warn people when you do update or something like that. I don't know how to look to warn them. And some interactions by email or something to see that there is, you know, some update or maintenance.” [12] |
| Additional feedback (13 respondents) | Disliked (3/13) | Negative overall experience (1) | “I guess, I, I actually felt very frustrated with the app, to be honest… Yeah, but it was like, I was looking forward to it, but I was just like, ‘oh, that's too bad.’ I, I, I, I was really, I was disappointed with it.” [2] |
|  |  | The users would not likely recommend the app to others (4) | “I haven't figured that out yet because I have talked about these ideas with quite a few friends, actually, over the years. Cause you know, it's been something I've been spending a lot of time on and you know, your friends ask you what you're doing, and then, um, and I've been looking for like a common thread amongst the people that accept these sorts of ideas more readily than others and I haven't found it yet. So, I, I don't have, I'm looking, I'm actively looking for that, *Chuckles* so you know, spare myself to trouble or trying to explain this to people that are never gonna bother to absorb it. But yeah, it almost seems like the people that need it the most are the most resistant.” [1]  “Um, it probably depends. Maybe someone who, like, just wanna know a bit more, they might use it initially, and then I don't know about them using it long term.” [13] |
|  |  | The users would not use this app to improve their PA (3) | “I feel bad saying that, but no, I wouldn't…” [2]  “Well, it's hard to say if, if it had some of the, I don't think it's, I mean, if you're using it as an educational tool, then you know, that's, that's different. I think I'm, I'm, my, my personal interest of having an app is about habit, is about recognizing the habits that I have and then, you know, making goals and seeing how much distance there is between where I am and where I wanna be. And then how to problem solve some of those things and how to change them and how many days it takes, you know, like if it was, oh, you know, it takes 21 days and you're five days away from making your habit stick or you know, something that kind of congratulates me for doing daily habit changing or awareness, even.” [4] |
|  | Liked (11/13) | Positive overall experience (11) | “I've enjoyed this process immensely. I found this very interesting and… I'm actually kinda, like, wanting the rest of the lessons *Chuckles*” [1]  “Very positive. It was very informative, easy to navigate, easy to use. It was a positive experience.” [7] |
|  |  | The users would likely recommend the app to others (7) | “Yeah, I think so. Yeah. Yeah, I think I can see people wanting to use it.” [3]  “I think that there's lots of, you know, there's, there's a few of these kind of apps that you can pay for right now, but, and, but people are always looking for ones that, that can, um, capture their needs. And if someone needed to actually create goals and also track, be able to track their active activity personally and also the, you know, number of steps I think is so awesome. Like, I think this would be helpful for them.” [5] |
|  |  | The users would use this app to improve their PA (6) | “Yes. I like apps like that. I like the validation of seeing a run streak. I, like, I have tons of apps like that for meditation and yoga, and I mean, like that in the sense that it gives you the reason why you should do it. It helps you think about how it fits into your life.” [1]  “Yes. I think I would, for sure. Especially I, yeah, I love the way that you could actually put in goals and achieve things like that. It, it appeals to me.” [5] |
| Additional features (11 respondents) | Suggestions (11/11) | Ability to take notes and add personal input throughout the modules (3) | “And, and maybe a, like, one thing I do, like, in habit apps that is motivating… is to see the little, you know, circles or boxes or something where I can see, ‘oh, I completed it on this date, I missed it on this date.’ And then to be able to add a note as to why I missed it. That is something I know I look for in the apps is if I can't do it that day, I wanna be able to add a note and say why.” [2]  “But that app also, it, you know, it was like you have to like link and sign up and it was like, I literally just want you to write down the numbers.” [4] |
|  |  | Ability to add comments about users' feelings and thoughts (1) | “Um, I know it's really about exercising, but maybe like a feelings check-in. Like how did I feel today? Like, did I, um, was I motivated? Was I not motivated, or did I really force myself to do this today or did I want to?” [10] |
|  |  | Ability to change goals everyday (1) | “Um, *Pause* I, I didn't, like, I needed to change the number of steps that I make per day. I can't make 10,000 steps, but I'm trying to increase from where I'm at, so I would have to change that every day instead of it remembering for me. And that is also something that I would love to have different.” [5] |
|  |  | Ability to connect to multiple tracking tools (1) | “And like I, I could connect it with other, I could connect my Google Fit with other apps, so, and I usually know how to do it, so I try different things, so, but I do like that option. Or if we could connect it to other apps maybe, or like Fitbits or different things, I feel like that would be good. Cuz then it's like, it's annoying if you use different apps and you have to input everything multiple times. So, I like having the, that all in one place.” [10] |
|  |  | Ability to put notes on the active minutes recorded to show what type of PA was performed (3) | “Uh, oh, the active minutes. I would love it if I could put what type of active minutes I did just, like, to look back and like, kind of help keep me going. Like, if I was like riding or walking fast or playing game or sports or whatever, like that's, that's, that would be something fun for me.” [5]  “Um, I don't know, maybe like being able to record what kind of activity you did. Yeah.” [11] |
|  |  | Ability to track blood pressure (2) | “The other things, some of the things I didn't get to use, but if you had the devices to track like your blood pressure or I guess, for example, things like that, that would be also helpful to have, too.” [9] |
|  |  | Add PA group lessons (1) | “Maybe, maybe you can add in this app, like it would be interesting to add some, you know, like in a gym, some lessons you do in gym, you know, group lessons.” [12] |
|  |  | Automatic syncing with Apple Health (1) | “Um, I liked that you had the option, especially with the steps to sync it with Apple Health and, like, it wasn't like it automatically synced. I do wish there was the option to also then set it to automatically sync after I knew kind of what I was getting into, and I prefer that for, I guess all of them instead of just the one. Cuz I found that I kept forgetting to enter it into one or the other. Yeah. I also like that, I guess when you synced it, it tells you if you went over and it gives you another green bar. So, I guess I keep going back and forth.” [11] |
|  |  | Add a baseline knowledge test (1) | “Another thing is that I noticed there wasn't, like, a baseline knowledge test, if that makes sense. Something I learned on, like, presentation skill, wanna do an air quote, like, is to have a baseline knowledge of the audience before introducing the content. And I think in this case it'll be really valuable to kinda have the, have the users, just, like, do a, do a mini quiz on what, how much they already know or at least what they're exposed to, just kind of get them to start thinking about it. And then, maybe at the end to test how much they learned throughout that. Cuz it'll be interesting even for themselves to track their progress and to feel that they actually, like, learn a lot.” [8] |
|  |  | Add incentives or show the users’ progress (3) | “…what I found that the app is lacking is some long-term, like, a reason to use this app long-term beyond the content part. But I, again, I may, maybe I would've said something different if it was a longer trial and I actually put in my steps to see graphs or putting my weight to see graphs. But, but that's something that my Fitbit does too, right? Yeah. I'm just trying to think about added value.” [7]  “But I think something like this, you could probably accumulate points and then, I don't know, go to, like, redeem it for something. That'd be pretty cool.” [13] |
|  |  | Daily notifications to improve user's motivation (1) | “I like, maybe like a daily one that might just pop up as a notification. Just kind to give, maybe give somebody the motivation to go. You wouldn't, I wouldn't want it to be like constant notifications where it's constantly making the phone go off. But even just once a day I think would help people.” [9] |
|  |  | Integrate problem-solving features to change users’ PA patterns (2) | “Um, yeah, I think if there was something that either popped up and asked you, you know, like a short-term goal and a long-term goal. Um, you know, maybe the short-term goal would be open this app every day and you know, maybe at the beginning of the day it says, ‘oh, I wanna take a 20-minute walk.’ And then in sort of solving that, and then being able to look back at your information like, it's kind of like a study and saying, you know, ‘oh, you know, this is, this is your excuse for the last four days is because you were tired’ or whatever. And then, you know, like those problem-solving things about, you know, well, you know, maybe your expectation, you know, just kind of some of that.” [4] |
